# Supplementary figures and images for: DNA barcoding and morphological identification of spiny lobsters in South Korean waters: a new record of Panulirus longipes and Panulirus homarus homarus
Source: PeerJ. 2022 Jan 10;10:e12744. doi: 10.7717/peerj.12744 (PMC8757375; doi:10.7717/peerj.12744)

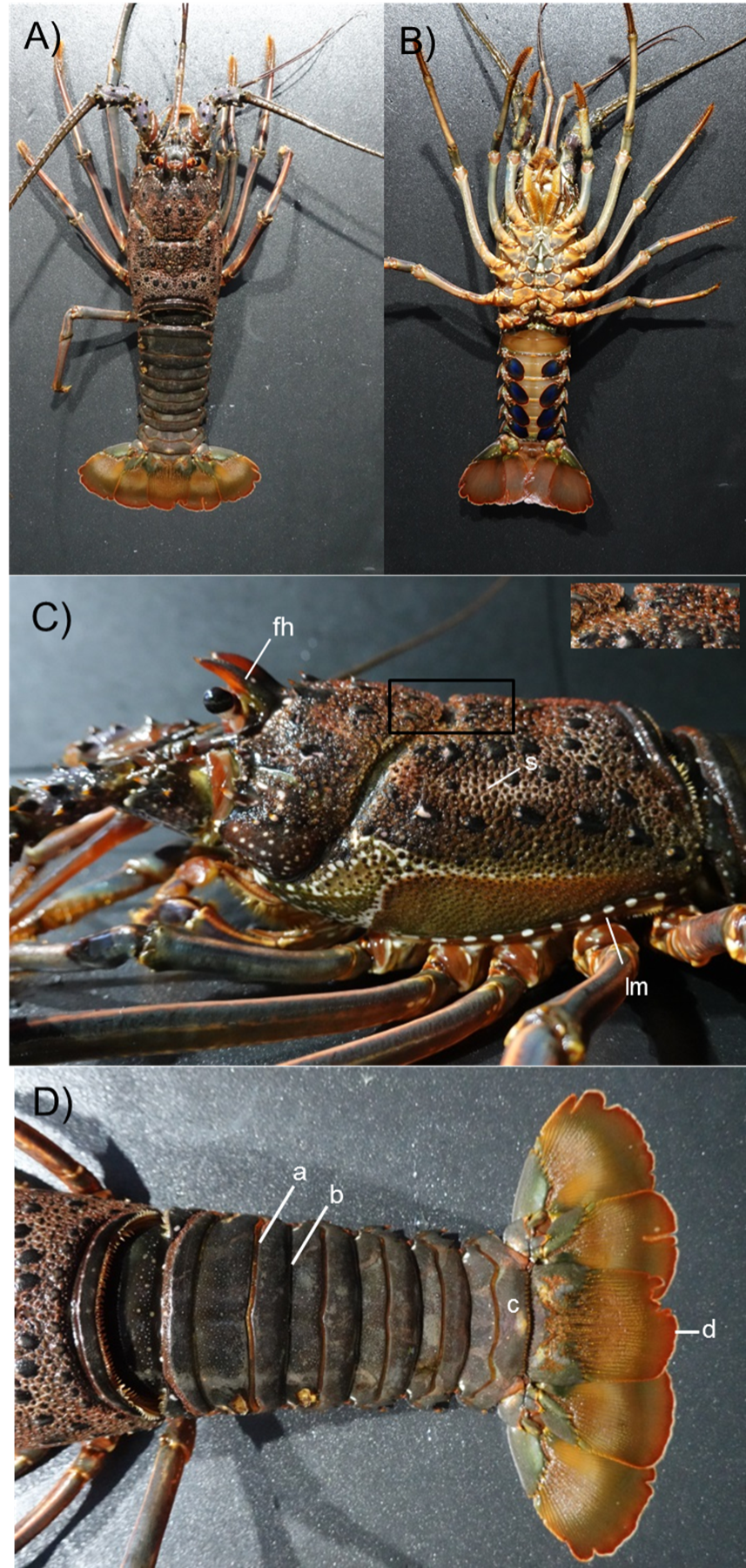

Supplement: Supplemental Information 1 — A) Dorsal side, B) Ventral side; C) Lateral side of the carapace; fh: Dark greenish brown frontal horns with white spots and orange color ventral margin; s: Randomly scattered spines on carapace with black color basal area; lm: White spotted lateral margin of the carapace; Enlarged area demarcated by rectangle: Reddish brown hairs on the mid-dorsal area of the carapace. D) Dorsal side of the abdomeinal somites; a: Non-interrupted transverse groove with posteriorly directed hairs; b: Posterior margin of the second somite with posteriorly directed hairs; c: Brown to purplish color base of telson; d: Reddish brown and slightly curved posterior margin of telson. [file peerj-10-12744-s001.png]

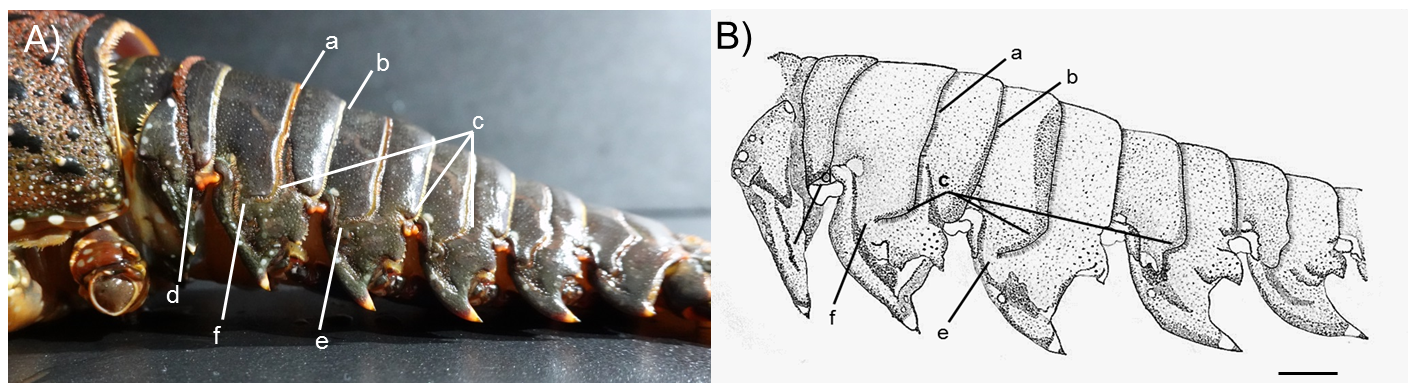

Supplement: Supplemental Information 2 — Arrows with a lowercase letter of photograph are indicating the following morphological features respectively; a: Posteriorly directed hairs in transverse groove; b: Posteriorly directed hairs in posterior margin of the second somite; c: Curved transverse groove at the lateral end of second, third and fourth somites; d: Interconnection between transverse groove and pleural groove at first somite; e: Gap between transverse groove and pleural groove at third somite; f: Gap between transverse groove and pleural groove at second somite. Scale bar represents two cm. [file peerj-10-12744-s002.png]
