# Supplementary material for: DNA barcoding and morphological identification of spiny lobsters in South Korean waters: a new record of Panulirus longipes and Panulirus homarus homarus
Source: PeerJ. 2022 Jan 10;10:e12744. doi: 10.7717/peerj.12744 (PMC8757375; doi:10.7717/peerj.12744)
Supplement: Supplemental Information 3 [file peerj-10-12744-s003.docx]

| No. | Sample ID | Scientific name | NCBI Accession No. | Location | Remarks |
| --- | --- | --- | --- | --- | --- |
| 1 | KSL1 | *P. japonicus* | OK037046 | Pyoseon Port | Jeju-type |
| 2 | KSL2 | *P. japonicus* | OK037047 | Pyoseon Port | Jeju-type |
| 3 | KSL3 | *P. japonicus* | OK037048 | Seogwipo Harbor | George & Holthuis (1965) |
| 4 | KSL4 | *P. japonicus* | OK037049 | Pyoseon Port | George & Holthuis (1965) |
| 5 | KSL6 | *P. japonicus* | OK037050 | Seogwipo Harbor | George & Holthuis (1965) |
| 6 | KSL7 | *P. japonicus* | OK037051 | Pyoseon Port | Jeju-type |
| 7 | KSL8 | *P. japonicus* | OK037052 | Pyoseon Port | George & Holthuis (1965) |
| 8 | KSL10 | *P. stimpsoni* | OK037053 | Seogwipo Harbor | George & Holthuis (1965) |
| 9 | KSL11 | *P. stimpsoni* | OK037054 | Seogwipo Harbor | George & Holthuis (1965) |
| 10 | KSL12 | *P. stimpsoni* | OK037055 | Seogwipo Harbor | George & Holthuis (1965) |
| 11 | KSL13 | *P. stimpsoni* | OK037056 | Seogwipo Harbor | George & Holthuis (1965) |
| 12 | KSL14 | *P. stimpsoni* | OK037057 | Seogwipo Harbor | George & Holthuis (1965) |
| 13 | KSL15 | *P. longipes* | OK037058 | Hwasun Harbor | George & Holthuis (1965) |
| 14 | KSL16 | *P. japonicus* | MZ203547 | Pyoseon Port | Jeju-type |
| 15 | KSL17 | *P. h. homarus* | MZ203548 | Hwasun Harbor | George & Holthuis (1965) |
| 16 | KSL18 | *P. longipes* | MZ203549 | Hwasun Harbor | George & Holthuis (1965) |
| 17 | KSL19 | *P. stimpsoni* | MZ203550 | Seogwipo Harbor | George & Holthuis (1965) |
